# Pre- and Postnatal Determinants of Deciduous Molar Hypomineralisation in 6-Year-Old Children. The Generation R Study

**DOI:** 10.1371/journal.pone.0091057

**Published:** 2014-07-02

**Authors:** Marlies E. C. Elfrink, Henriette A. Moll, Jessica C. Kiefte-de Jong, Vincent W. V. Jaddoe, Albert Hofman, Jacob M. ten Cate, Jaap S. J. Veerkamp

**Affiliations:** 1 Department of Cariology, Endodontology and Pedodontology, Academic Centre for Dentistry Amsterdam (ACTA), University of Amsterdam and VU University Amsterdam, Amsterdam, The Netherlands; 2 Department of Pediatrics, Erasmus Medical Centre - Sophia Children's Hospital, Rotterdam, The Netherlands; 3 The Generation R Study Group Erasmus Medical Centre - Sophia Children's Hospital, Rotterdam, The Netherlands; 4 Department of Epidemiology, Erasmus Medical Centre - Sophia Children's Hospital, Rotterdam, The Netherlands; University of North Carolina at Chapel Hill, United States of America

## Abstract

**Background:**

Deciduous Molar Hypomineralisation (DMH) and Molar Incisor Hypomineralisation (MIH) are common developmental disturbances in pediatric dentistry. Their occurrence is related. The same determinants as suggested for MIH are expected for DMH, though somewhat earlier in life. Perinatal medical problems may influence the prevalence of DMH but this has not been studied sufficiently.

**Objective:**

This study aimed to identify possible determinants of DMH in a prospective cohort study among 6-year-old children.

**Study Design:**

This study was embedded in the Generation R Study, a population-based prospective cohort study from fetal life until young adulthood. The the data were used to identify the determinants of DMH. Clinical photographs of clean, moist teeth were taken with an intra-oral camera in 6690 children (mean age 6.2 years; 49.9% girls). Data on possible determinants that had occurred during pregnancy and/or the child's first year of life were on the basis of manual standardized measurements (like length and weight) and questionnaires. Multivariate analyse with backward and forward selection was performed.

**Results:**

A number of factors in the pre-, peri- and postnatal phase were found to be associated with DMH. After multivariate logistic regression analyses, Dutch ethnic background, low birth weight, maternal alcohol consumption during pregnancy, and fever episodes in the first year of the child's life were found to play a role in the development of DMH in 6-year-old children.

**Conclusion:**

This study shows that Dutch ethnicity, low birth weight, alcohol consumption by the mother during pregnancy and any fever in the first year of the child's life are associated with DMH. Not only childhood factors but also prenatal lifestyle factors need to be taken into account when studying determinants for DMH.

## Introduction

Deciduous Molar Hypomineralisation (DMH) is a recently identified hypomineralisation disturbance in the enamel of the deciduous dentition with varying prevalence in 1–4 second primary molars. The enamel hypomineralisations in DMH are similar to those observed in Molar Incisor Hypomineralisation (MIH) in the permanent dentition [1]. Molar Incisor Hypomineralisation (MIH) is a hypomineralisation disturbance of the enamel of 1–4 first permanent molars sometimes in combination with hypomineralised incisors [2]. DMH and MIH are common developmental disturbances of the enamel [3]. The prevalence of DMH varied between 4.9%–9.0% [1], [4], [5]. Studies on MIH worldwide showed prevalence-rates between 2.4% and 40.2% and the most recently published prevalence in the Netherlands of MIH was 14.3% [3], [6]. Children with DMH had an increased risk to develop MIH as well (OR 4.4, 95%CI: 3.1–6.4) [4]. Because the second primary molars erupt four years earlier in life than the first permanent molars, DMH is a clinically useful predictor for MIH [4]. Due to the enamel hypomineralisation, MIH and DMH teeth are more prone to caries [7], [8]. MIH causes discomfort for the child, even without caries since the affected teeth are reported to be very sensitive for cold and heat [8], the same seems to be the case for DMH.

For DMH and MIH, the same determinants have been suggested, although occurring somewhat earlier in life for DMH than for MIH [1], [9]. Because the development of the primary teeth is earlier than the development of the permanent teeth, the possible determinants need to have occurred be earlier in life. Some recent reviews on MIH focus on possible determinants: medical problems in the prenatal, perinatal and postnatal period, medicine use of the child and exposure to environmental pollution during the first years of life [10], [11]. Several factors have been identified as determinants for MIH, but the conclusions of these studies were sometimes contradictory especially regarding medication use, feeding and illnesses [10], [11], [12]. Animal experiments on fever, dioxin exposure and the use of antibiotics (especially amoxicillin) showed these factors influence the enamel formation and may cause enamel hypomineralisation [11], [13], [14], [15], [16]. No relation between DMH and the use of antibiotics, anti-allergic medicines or anti-asthmatic medicines of the mother during pregnancy was found [17].

Possible determinants of DMH have been hypothesised on. Medical problems in the perinatal period were found to be related to the occurrence of DMH, but no specific determinants are yet identified [9]. All proposed determinants in this study were based on potential determinants of MIH. Although pre- and perinatal factors do not seem to have much influence on MIH [11], [18], they do seem to play an important role in DMH [9]. On the other hand, factors associated to DMH might be indicative for future development of MIH.

Therefore, the aim of this study was to identify prenatal, perinatal and early postnatal determinants of DMH using a large prospective cohort study.

## Materials and Methods

### Ethics statement

The study was approved by the Medical Ethics Committee of the Erasmus Medical Centre, Rotterdam, and was conducted in accordance with the declaration of Helsinki. All parents/caretakers gave written informed consent on behalf of themselves and their children [19].

### Participants

This study was embedded in the Generation R Study, previously described in detail [19], [20]. This population-based prospective cohort study from fetal life until young adulthood was designed to identify early environmental and genetic determinants of growth, development and health. At enrolment, the cohort included 9778 mothers and their children living in Rotterdam, the Netherlands. All children were born between April 2002 and January 2006 and formed a prenatally enrolled birth-cohort. Of all eligible children in the study area, 61% participated at birth in the study [19]. For the postnatal phase of the study, 7893 children were available.

**Figure 1:**
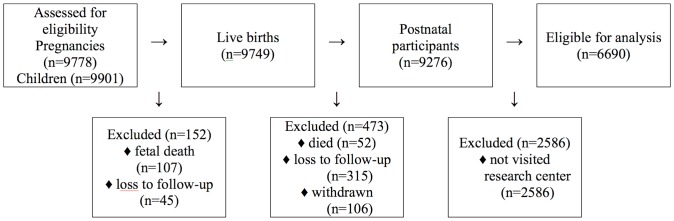
Flowchart of the participants.

From March 2008 until January 2012, 6690 6-year-old children, including 88 twins, visited the Erasmus Medical Centre. A flowchart of the participants is shown in Figure 1.

### Measurements

Assessments were planned in early pregnancy (gestational age <18 weeks), mid pregnancy (gestational age 18–25 weeks) and late pregnancy (gestational age >25 weeks) and included questionnaires on lifestyle and general health, physical examinations and fetal ultrasound examinations. Postnatal information on the growth, development and health of the participating children at the ages of 2, 6 and 12 months was obtained from manual measurements at the routine child health centres and by questionnaires. Apgar scores and weight and length at birth were available from delivery reports. Ethnicity [21], education level [22], household income, additional use of folic acid, and the health of the mother and child, were collected via frequently distributed questionnaires [19].

Ethnicity was based on the country of birth of the parents. If both parents were born in the Netherlands, the ethnicity was considered Dutch. The non-Dutch ethnicity was divided into ethnic backgrounds: Turkish, Moroccan, Surinamese and other. The country of birth of the mother determined the ethnic background [23].

At the age of 6 years, children visited the research centre for manual measurements and to have photographs taken of their teeth. After brushing their teeth, photographs of clean, moist teeth were successfully taken in 6325 children (94.5%).

Trained nurses and dental students took approximately ten photographs of all the teeth within 1–2 minutes per child. An intra-oral camera (Poscam USB intra-oral autofocus camera [Digital Leader PointNix] or SOPRO 717 intra-oral autofocus camera [Acteon], 640×480 pixels) were used for the photographs, with a minimal scene illumination of f 1.4 and 30 lx. In The validity of an intra-oral camera for visualizing DMH had been shown to be high [24].

DMH was scored from the intra-oral photographs using the EAPD criteria [2], [24], [25]:

**Opacity.** A defect that changes the translucency of the enamel, variable in degree. The defective enamel is of normal thickness with a smooth surface and can be white, yellow or brown in color. The demarcated opacity is not caused by caries, ingestion of excess fluoride during tooth development or amelogenesis imperfecta etc., and/or
**Post-eruptive enamel loss.** A defect that indicates surface enamel loss after eruption of the tooth, e.g. hypomineralisation related attrition. Enamel loss due to erosion was excluded, and/or
**Atypical caries.** The size and form of the caries lesion do not match the present caries distribution in the child's mouth, and/or
**Atypical restoration.** The size and form of the restoration do not match the present caries distribution in the child's mouth, and/or
**Atypical extraction.** Absence of a 2^nd^ primary molar that does not fit in the dental development and caries pattern of the child.

When at least one of these criteria was fulfilled, a second primary molar was diagnosed as having DMH. In cases in which a few teeth could not be scored, only the teeth visible on the photographs were used in the analysis. If the tooth, or the place where the tooth should be, did not show on the photographs, the tooth was scored as ‘not able to be judged’. Also partial visible teeth, teeth covered with debris or saliva or photographs of low quality were scored as ‘not able to be judged’.

The photographs were displayed on a computer in full-screen mode and scored by a single calibrated dentist (ME). To test the inter-observer agreement in this study, the data of 648 children were scored independently by another calibrated dentist (JV). Calibration was repeated regularly during the research period. The Cohen's kappa score in this study was 0.73 for DMH. In the event of a disagreement, the photographs were studied again, and a consensus decision was made. A separate group of 649 children were scored again by the first dentist (ME), at least six weeks after the first scoring. The intra-observer agreement reached Cohen's kappa scores of 0.82 for DMH.

### Statistics

Statistical analyses were performed with SPSS version 18.0 (SPSS Inc, Chicago, IL, USA). To identify determinants of DMH, logistic regression analysis was used. Univariate logistic regression models were performed to assess the associations of the putative determinants with DMH. A list of all used putative determinants is shown in Table 1. Subsequently, all putative determinants with a p-value<0.20 (15 factors, see Table 2) were simultaneously included in a multivariable model. In order to identify the most significant independent determinants, this multivariable logistic regression analysis was performed using backward and forward selection procedures retaining only the strongest determinants of DMH with p = 0.05 as endpoint. A multiple imputation procedure was used (n = 10 imputations) to complete the data from the 6690 children [26]. The imputations were repeated for 10 times and the data were imputed according to the Markov Chain Monte Carlo (MCMC) method (assuming no monotone missing pattern). In each data set the data were separately analysed and the results of the 10 imputed analyses were pooled. In this paper only the original data were reported because the results on the original data were not significantly different from the imputed data. The eligible data vary per determinant due to missing data. A p-value<0.05 was considered as statistically significant.

**Table 1:** Putative determinants used in univariate logistic regression analysis.

	Lifestyle	Environmental	Health
**Prenatal**	Ethnicity child, Education level mother, Household income, Smoking mother, Additional use folic acid, Maternal alcohol consumption during pregnancy	Air pollution, Possibly hazardous substances working environment mother (like agricultural chemical, glue, cleaning agents, metals, radiation (x-ray, UV))	Medication use mother, Illnesses (fever, flu), Vomitting and diarrhea, Pregnancy induced diabetes, High bloodpressure because of pregnancy
**Perinatal**	-	Twin pregnancy	Birthweight/LBW, Apgar 1&5 min, Pre-eclampsia (HELLP), IUGR (intra-uterine growth retardation), Small for gestational age, Hospitalisation 1st week of life
**Postnatal**	Breastfeeding (6 months), Additional vitamin D, Introduction foods (6 months)	Medication use mother during breastfeeding (from questionnaires)	Medication use child (from questionnaires), Fever episodes, Illnesses, Shortness of breath/wheezing, Vomitting and diarrhea

**Table 2:** Odds ratios for possible determinants for DMH after univariate logistic regression.

			Children without DMH (n = 5183)		Children with DMH (n = 515)			
			n	%	n	%	OR	95% CI
**Prenatal**	Lifestyle	Ethnicity child						
		*Dutch*	3175	61.3	388	75.3	Ref	
		*Turkish*	349	6.7	20	3.9	0.47 **	0.30–0.75
		*Moroccan*	268	5.2	22	4.3	0.67 #	0.43–1.05
		*Surinamese*	359	6.9	23	4.5	0.52 *	0.34–0.81
		*Other ethnicity (Cape Verdean, Antillean, Asian etc)*	685	13.2	36	7.0	0.43 **	0.30–0.61
		Education level mother						
		*Primary education*	430	8.3	23	4.5	Ref	
		*Secondary education*	2076	40.1	189	36.7	1.70 *	1.09–2.66
		*Higher education*	2207	42.6	266	51.7	2.25 **	1.45–3.49
		Household income (per month)						
		*<2200 euro*	1651	31.9	129	25.0	Ref	
		*>2200 euro*	2252	43.5	282	54.8	1.60 **	1.29–1.99
		Additional use folic acid						
		*No*	865	16.7	72	14.0	Ref	
		*Start first 10 weeks*	1121	21.6	133	25.8	1.43 *	1.06–1.92
		*Start periconceptional*	1515	29.2	169	32.8	1.34 *	1.01–1.79
		Maternal alcohol consumption during pregnancy						
		*No*	1874	36.2	143	27.8	Ref	
		*Yes*	2168	41.8	272	52.8	1.64 **	1.33–2.03
	Health	Vomitting and diarrhea						
		*No*	1637	31.6	177	34.4	Ref	
		*Yes*	2925	56.4	272	52.8	0.86 #	0.71–1.05
**Perinatal**	Health	Low Birth Weight (<2500 g)						
		*No*	4886	94.3	474	92.0	Ref	
		*Yes*	287	5.5	40	7.8	1.44 *	1.02–2.03
		Small for Gestation Age (<2 z-score)						
		*No*	4512	87.1	430	83.5	Ref	
		*Yes*	66	1.3	13	2.5	2.07*	1.13–3.78
		Apgar score 1 minute						
		*≥7*	4302	83.0	433	84.1	Ref	
		*<7*	281	5.4	20	3.9	0.71 #	0.45–1.13
		Apgar score 5 minute						
		*≥7*	4802	92.7	482	93.6	Ref	
		*<7*	56	1.1	2	0.4	0.36 #	0.09–1.46
		Hospitalisation first week of life						
		*No*	2609	50.3	266	51.7	Ref	
		*Yes*	533	10.3	68	13.2	1.25 #	0.94–1.66
**Postnatal**	Lifestyle	Breastfeeding at 6 months						
		*No*	2438	47.0	290	56.3	Ref	
		*Yes*	1191	23.0	112	21.7	0.79*	0.63–0.99
	Health	Antibiotic use child first year						
		*No*	2237	43.2	222	43.1	Ref	
		*Yes*	1495	28.8	178	34.6	1.20 #	0.98–1.48
		Fever episodes in first year						
		*No*	652	12.6	47	9.1	Ref	
		*Yes*	3100	59.8	356	69.1	1.59 **	1.16–2.18
		Vomitting and diarrhea						
		*No*	1802	34.8	169	32.8	Ref	
		*Yes*	1936	37.4	234	45.4	1.29 *	1.05–1.59

(# p≤0.20, * p≤0.05, ** p≤0.01).

## Results

From the 6690 participating children, a complete set of photographs was made in 94.5%. Only one photograph was taken in 3.2% and no photographs were taken in 2.3% of the children. In this study, the data from 6325 children were used (mean±SD age 6.2±0.53 years; 49.9% girls). In 5697 children presence or absence of DMH could be established. For the remaining children, the drop-out was mostly due to limitations in scoring individual teeth. The prevalence of DMH was 9.0% (n = 515) at the child level. Of all eligible second primary molars (n = 24347), DMH was present in 4.1% (n = 987). Often children only had one molar affected, and the mean (±SD) number of DMH molars per child was 1.9 (±0.99).

A number of determinants, based on the possible determinants for MIH, with some prenatal factors added, were analysed. These determinants encompassed prenatal lifestyle and health factors like ethnicity, education level, household income, use of folic acid during pregnancy, use of alcohol during pregnancy, vomiting & diarrhea (mother), low birth weight and small for gestational age; perinatal factors like apgar scores and hospitalisation in the first week of life; and postnatal lifestyle and health factors like breastfeeding at 6 months, antibiotic use, fever episodes and vomiting & diarrhea (child). All putative determinants with a p-value<0.20 are shown in Table 2.

Dutch ethnicity, alcohol consumption by the mother during pregnancy, low birth weight and fever episodes in the first year of life were identified as determinants for DMH (Table 3). Results were not significantly different after the multiple imputation procedure (data not shown).

**Table 3:** Final multivariate model after backward and forward selection procedures in logistic regression analysis.

Determinants	p-value	OR	95%CI
Ethnicity (Dutch vs Turkish)	0.035	0.49	0.25–0.95
Ethnicity (Dutch vs Moroccan)	0.290	0.68	0.34–1.39
Ethnicity (Dutch vs Surinamese)	0.046	0.56	0.32–0.99
Ethnicity (Dutch vs “other ethnicity”)	<0.001	0.45	0.28–0.70
Low Birth Weight	0.007	1.91	1.19–3.05
Maternal alcohol consumption during pregnancy	0.013	1.39	1.07–1.80
Fever episodes in the child first year of life	0.035	1.48	1.03–2.12

Odds ratios and p-values for the determinants are given.

## Discussion

This study adds new determinants for the developmental disturbance DMH. Ethnicity, alcohol consumption by the mother during pregnancy, low birth weight and any fever in the first year of life were found to be associated with DMH. These results are partly in line with conclusions based on MIH research. However, we note that the underlying causative mechanism could be genetic but also environmentally oriented. The second primary molar and first permanent molar have a shared period of development and mineralisation, and an observed relationship between DMH and MIH had already been hypothesised upon [2], [4]. The development of the second primary molar and first permanent molar start at the same moment in time, but the maturation phase of the permanent molar is considerably longer [27]. If a risk factor occurred during this overlapping period, enamel hypomineralisation might occur in both the primary and permanent dentition [28]. The determinants for DMH are expected to be more pre- and perinatal than postnatal [9]. Beentjes et al. suggested that MIH is caused by a combination of factors and/or a the factors need to reach a threshold level before enamel defects are being caused [10], [11], [29], [30]. A comparable explanation is probably the case for DMH, as several, commonly occurring factors were found as determinants for DMH.

Some of the variables that showed statistical significance in the univariable analyses disappeared after correction for confounding variables like SES. In other studies, the additional factors folic acid use, ethnicity and maternal alcohol consumption were also related to a higher SES [31], [32]. We therefore conclude that lifestyle/social economic status factors are a major determinant for the occurrence of DMH. There are no studies yet on the relationship between MIH or DMH and SES.

Dutch ethnicity and alcohol consumption were not mentioned previously in MIH research. Most studies on MIH and DMH have been performed in northern Europe, probably because enamel hypomineralisations were seen most often in those countries [3]. The Caucasian background may cause a lower threshold for DMH and MIH. Hargreaves et al [33] did find differences between ethnical groups in South Africa. They found less hypoplasia and hypocalcification in the primary dentition in the white children [33]. Probably lifestyle influences influence the outcome here.

Most studies were not performed in a large multi-ethnic cohort, like the Generation R project; therefore the influence of ethnicity could not be studied. Studies on varying prevalences of MIH from other parts of the world are still being published [3], [34], [35], [36], [37].

Possible causes of enamel hypomineralisation were also studied in animal research. For example, animal research has shown that ethanol (alcohol) can lead to changes in cellular differentiation and enamel mineralisation [38]. Our observed association of alcohol consumption during pregnancy with enamel hypomineralisation has not been reported before and needs further exploration. Studying dose related effects of alcohol on DMH was not possible due to small numbers of the high exposure category [39] and requires additional studies.

Low birth weight children seemed to be at greater risk for enamel defects in the primary dentition than children with normal birth weight [40], [41]. For MIH, low birth weight does not seem to be a determinant. Low birth weight might be associated with DMH, but caution should be taken since the study from Vello et al. [40] and Rugg-Gunn et al. [41] was based upon another index (modified Developmental Defects of Enamel (mDDE)) for scoring the enamel defects in which enamel defects on all primary teeth were taken into account. Low birth weight is likely to interact with other possible determinants related to maternal health status for enamel defects.

Fever is often mentioned in MIH research [11] as a possible determinant. In an animal study, enamel hypomineralisation of incisors could be induced by fever in rats [13]. Cells related to the formation of the enamel prisms were thought to be affected by fever, thereby causing the enamel hypomineralisation [13]. The present study shows that fever in the first year of life is one of the determinants of DMH, so febrile infectious diseases might play a role.

The cause of DMH seems to be related to general perinatal morbidity, but some determinants were previously mentioned determinants in MIH research. This observation supports the earlier finding of a direct relationship between DMH and MIH [4] and emphasises the prospective nature of DMH for occurrence of MIH.

A strength of the present study is the unselected population and prospective study design. By comparison, most earlier studies were small, selected and retrospective, presenting biased data since parents were likely not be able to accurately remember details that happened about eight years before [10], [11], [12]. In this study the questionnaires were filled out every 3 months in the prenatal phase and for children at the age of 2, 6 and 12 months [19].

To appreciate the results also some limitations of the study need to be discussed. Data collected by means of questionnaires (e.g. use of folic acid, alcohol consumption, education level, household income, breastfeeding and fever) are probably less reliable. Bias can occur because parents give answers they think are the best answers or they forget some incidentally happening events (e.g. fever). With the use of repeated questionnaires during pregnancy and the first year of life, the change of forgetting to fill out incidentally happening events is decreased.

The proportion of mothers with different ethnicities and lower socio-economic statuses were lower among the participants than expected from the population data in the study area [19]. The selection towards a more affluent and healthier population might influence the generalisability of the results but can only reinforce the results of this study. Therefore, we do not anticipate that the association between the identified determinants and DMH would be different in the participating population compared to the non-participating population.

Taking the photographs was difficult in some of the young children. Unsuccessful pictures were generally seen in cases in which the child was not able to breathe nasally, e.g., due to a common cold, thus creating moisture on the lens of the camera. Due to the small number of missing photographs and the fact that they are missing at random, the results are still considered representative.

The determinants found in this study all seem to be related to disturbances in a child's physical development. Teeth and more specifically enamel can contain signs of this process. More research is needed to explain the interactions between determinants and investigate possible severity-related effects.

## Conclusion

This study shows that Dutch ethnicity, low birth weight, alcohol consumption by the mother during pregnancy and any fever in the first year of the child's life are associated with DMH. Therefore not only childhood factors but also prenatal factors need to be taken into account when studying determinants for DMH.
